# A Novel Antisense RNA from the *Salmonella* Virulence Plasmid pSLT Expressed by Non-Growing Bacteria inside Eukaryotic Cells

**DOI:** 10.1371/journal.pone.0077939

**Published:** 2013-10-31

**Authors:** Jesús Gonzalo-Asensio, Álvaro D. Ortega, Gadea Rico-Pérez, M. Graciela Pucciarelli, Francisco García-del Portillo

**Affiliations:** 1 Centro Nacional de Biotecnología, Consejo Superior de Investigaciones Científicas (CNB-CSIC), Madrid, Spain; 2 Departamento de Biología Molecular, Universidad Autónoma de Madrid. Centro de Biología Molecular ‘Severo Ochoa’ (CBMSO-CSIC), Madrid, Spain; Indian Institute of Science, India

## Abstract

Bacterial small RNAs (sRNAs) are regulatory molecules playing relevant roles in response to environmental changes, stressful conditions and pathogenesis. The intracellular bacterial pathogen *Salmonella enterica* serovar Typhimurium (*S.* Typhimurium) is known to regulate expression of some sRNAs during colonization of fibroblasts. Here, we characterize a previously unknown sRNA encoded in the *S.* Typhimurium pSLT virulence plasmid that is specifically up-regulated by non-growing dormant bacteria persisting inside fibroblasts. This sRNA was inferred in microarray expression analyses, which unraveled enhanced transcriptional activity in the *PSLT047- PSLT046* (*mig5*) intergenic region. The sRNA transcript was further identified as a 597-nucleotide molecule, which we named IesR-1, for ‘Intracellular-expressed-sRNA-1′. IesR-1 expression is low in bacteria growing in axenic cultures across a variety of experimental conditions but displays a marked increase (∼200–300 fold) following bacterial entry into fibroblasts. Remarkably, induction of IesR-1 expression is not prominent in bacteria proliferating within epithelial cells. IesR-1 deletion affects the control of bacterial growth in defined fibroblast cell lines and impairs virulence in a mouse infection model. Expression analyses performed in the *PSLT047-iesR-1*-*PSLT046* (*mig5*) region support a *cis*-acting regulatory mechanism of IesR-1 as antisense RNA over the *PSLT047* transcript involving interaction at their respective 3′ ends and modulation of PSLT047 protein levels. This model is sustained by the scarce production of PSLT047 protein observed in non-growing intracellular bacteria and the high amount of PSLT047 protein produced by bacteria carrying a truncated IesR-1 version with separated 5′ and 3′ regions. Taken together, these data reveal that *S.* Typhimurium sRNAs encoded in the pSLT virulence plasmid respond to a state of persistence inside the host cell. As exemplified by IesR-1, some of these sRNAs may contribute to diminish the relative levels of proteins, such as PSLT047, which are probably dispensable for the intracellular lifestyle.

## Introduction

Small non-coding RNAs (sRNAs) constitute a family of molecules widely distributed in prokaryotes that has emerged as important elements in regulatory circuits [1]–[3]. sRNAs are 50–400 nucleotides in length, most often expressed as transcriptional units from intergenic regions (IGR) and include a structured stem-loop Rho-independent terminator at the 3′ end. Computational algorithms designed for sRNA discovery in bacteria have mainly relied on the search within IGRs for conserved, relatively short sequences, flanked by orphan promoters and Rho-independent terminators [4]. Direct cloning of small RNAs and high-density microarray and RNA-seq approaches have unraveled the sRNAome of different bacterial species, including pathogens [5]–[9]. sRNAs can interact to either nucleic acid-binding proteins modulating their regulatory activity by titration or to target mRNAs by direct base-pairing complementarity. The latter include *cis*-acting sRNAs, codified in the same genetic region but in antisense orientation to their target mRNAs and *trans*-acting sRNAs, which are transcribed elsewhere in the chromosome and require the RNA chaperone Hfq to assist sRNA binding to multiple mRNA targets. Target sites in *trans*-regulated mRNAs have been most often found in the 5′ untranslated region (UTR) of the transcript. This binding results in the modulation (positive or negative) of the mRNA stability and/or translation [1].

sRNAs have been identified in intracellular bacterial pathogens such as Salmonella enterica, Legionella pneumophila, Listeria monocytogenes, Yersinia spp., Streptococcus pneumoniae and Mycobacterium tuberculosis, among others [8], [10]–[15]. These pathogens reprogram the transcriptome/proteome to successfully survive in the harsh environment encountered within host cells. Transcriptional reprogramming involves adaptive mechanisms, including adjustment of the sRNAs levels. A recent study has shown increased expression of defined sRNAs in S. enterica serovar Typhimurium (S. Typhimurium) when this pathogen invade fibroblasts, a host cell type in which intracellular bacteria establish a non-proliferative state [16]. Induction of sRNAs by intracellular S. Typhimurium has also been reported in macrophage-like cells, as it is the case of OxyS [17], an sRNA previously shown to be up-regulated in response to oxidative stress [18]. Although most mutants lacking defined sRNAs often show mild phenotypes, a few sRNA-defective mutants display impaired virulence in vivo [11]–[13], [15], [19]. Mutants lacking IsrM are impaired for invasion of epithelial cells and exhibit reduced proliferation in macrophage-like cells. The absence of IsrM also affects the capacity of S. Typhimurium to growth in mouse organs [11]. It is also known that mutants defective in IstR or SroA exhibit reduced fitness in mice [15]. Mechanistically, the sRNA IsrM affects post-transcriptionally the expression of the virulence factor HilE and the secreted effector SopA [11]. Decreased translocation of the protein effector SptP into epithelial cells was also linked to a defect in the sRNA IsrJ [17]. Whether additional S. Typhimurium sRNAs contribute to other aspects of the host-pathogen interplay, such as that occurring during non-proliferative persistent infections, is at present unknown.

Some pathogens have developed strategies to undergo a persistent infection in specific host cell types, which allow them to evade host immune responses [20]. In humans, some intracellular pathogens as *S. enterica* serovar Typhi, *Helicobacter pylori* and *M. tuberculosis,* are prone to cause this type of infections [21]. *S.* Typhimurium is a suitable model for persistent infection given the ability of this pathogen to cause chronic infections in resistant mice [22]. Recent work has shown that *S*. Typhimurium restrains growth *in vivo* within non-phagocytic cells positioned in the intestinal *lamina propria* of susceptible mice and that such phenotype is reproduced in primary intestinal fibroblasts [23]. Upon invasion of cultured fibroblasts, most intracellular bacteria actively attenuate growth, a response that requires a subset of pathogen regulators including the PhoP-PhoQ system [23], [24]. Recently, we analyzed in *S.* Typhimurium the expression of chromosomally-encoded sRNAs in non-proliferating bacteria persisting within fibroblasts [16]. This study demonstrated that some sRNAs are specifically expressed along the intracellular infection process. Whether some of these sRNA play a dedicated role in modulating growth of the pathogen inside the host cell has not been yet interrogated.

The genome of the *S.* Typhimurium virulent strain SL1344 comprises a 4.9 Mb chromosome and a 94 Kb virulence plasmid named pSLT [6], [25]. Genetic mobile elements, including plasmids, frequently comprise non-coding RNA loci [26]. An example is the *hok/sok* type I toxin-antitoxin system involved in the post-segregational killing mechanism employed by the R1 plasmid in *E. coli* [27]. Conjugal transfer of pSLT is also controlled by a *cis*-acting sRNA, namely FinP, which negatively regulates translation of the adjacent *traJ* gene [28], [29]. Most studies on sRNAs have been however conducted in the chromosome, overlooking the possible presence of these molecules in the virulence plasmid. A notable exception was a comprehensive RNAseq study focused in deciphering the transcriptional map of *Salmonella* plasmids during the early stationary phase of growth [6]. The expression of these RNAs during infection conditions was, however, not examined.

In this work, we searched for novel sRNAs in the *Salmonella* virulence plasmid pSLT using microarray data obtained from intracellular bacteria that colonize fibroblasts. We selected a novel sRNA exhibiting significant expression in non-growing intracellular wild type bacteria. Further analysis of the locus displaying such transcriptional activity revealed the presence of an antisense sRNA positioned between *PSLT047* and *PSLT046* (*mig5*) genes. The expression of this sRNA, which we named IesR-1, for ‘Intracellular-expressed-sRNA-1′, was low in most of the laboratory growth conditions tested while increased dramatically in non-growing intracellular bacteria. Manipulation of the relative levels of IesR-1 also led to alteration of virulence in the mouse typhoid model. Collectively, the data obtained in this work support a model involving a *cis*-acting mechanism of IesR-1 over *PSLT047* with interaction at their respective 3′ ends, a phenomenon that could modulate PSLT047 translation. This mechanism is consistent with the marked decrease of the PSLT047 protein observed in intracellular bacteria.

## Materials and Methods

### Ethics Statement

Animal research adhered to the principles mandatory in the European Union, as established in the Legislative Act 86/609 CEE (November 24, 1986), and followed the specific protocols established by the Royal Decree 1201/2005 of the Government of Spain (October 10, 2005). The protocols employed in the study were reviewed by the ‘Comité Ético de Experimentación of the Consejo Superior de Investigaciones Científicas (CSIC)’ and were approved on March 15, 2007.

### Bacterial Strains and Growth Conditions

The *S. enterica* serovar Typhimurium wild-type strain SV5015 (SL1344 His^+^) [30] and its isogenic mutant MD1120 (*phoP7953::*Tn10) [24], were used. The strain EG5510 (*phoP*-*24*) carrying a constitutive active allele was a gift from Eduardo A. Groisman (Yale University). The MD2218 (Δ*iesR-1*/5′::*cat*) strain, derived from SV5015, was constructed using the one-step inactivation procedure [31]. Amplification products were obtained from plasmid pKD3 as a template using primers KO-995-fw and KO-995-rv (Table S1). The antibiotic resistance gene from MD2218 was eliminated by an FLP recombinase, encoded in plasmid pCP20 [31], resulting in strain MD2219 (Δ*iesR-1*/5′). The strain MD2255 (*PSLT047*::3xFLAG-Km) was constructed by the method described in [32] using primers FLAG-PSLT047-fw and FLAG-PSLT047-rv (Table S1). The kanamycin resistance marker was eliminated using the pCP20-encoded FLP recombinase to obtain the strain MD2256 (*PSLT047*::3xFLAG).

The MD2219 (Δ*iesR-1*/5′) strain was used as recipient in genetic procedures designed to complement the defect in IesR-1. Two versions for complementation purposes were generated, one carrying the full-length IesR-1molecule (‘long’ variant) and the second, 208 nt of the 5′ region (‘short’ variant). The procedure was based in the usage of the PLtetO promoter [33] to drive the expression of the RNA molecule of interest. The construction of the ‘long’ and ‘short’ versions of IesR-1 and their subsequent integration in the chromosomal locus *araE* involved the following steps: i) insertion of a CAT-PLtetO locus in the promoter region of *iesR-1* by the one-step method described by Datsenko and Wanner [31]; ii) PCR-amplification of the CAT-PLtetO-*iesR-1/5′* (short) or CAT-PLtetO-*iesR* (‘long’) constructs with ends containing *araE* sequences; and, iii) insertion of the PCR fragments in the *araE* locus by one-step method using chloramphenicol resistance as marker. The chloramphenicol-resistance cassette (CAT) and the PLtetO promoter sequences were in divergent orientations in all the constructions. The production of the “short” (*iesR-1/5′*) and “long” (complete *iesR-1*) molecules was confirmed by RT-PCR. The genotype of the resulting strains was: MD2276: Δ*iesR-1/*5′ CAT::PLtetO *iesR-1*/5′; MD2277: Δ*iesR*-1/5′, CAT::PLtetO *iesR-1*. The oligonucleotides used in these procedures are listed in Table S1.

Bacteria were grown routinely in Luria Bertani (LB) broth. When required bacteria were cultured in PCN defined medium at pH 5.8 [34] or in ISM medium [35]. Incubation was performed at 37°C in non-shaking or shaking (180 rpm) conditions. When appropriate, tetracycline (10 µg/ml), kanamycin (30 µg/ml) or chloramphenicol (10 µg/ml) were added to the growth medium.

### Total RNA Isolation and cDNA Synthesis

Cultures were inoculated with overnight-grown bacteria (dilution 1∶100, OD∼0.02). Standing cultures were incubated overnight. Shaking cultures were incubated at 180 rpm until early-exponential phase (OD_600_ ∼ 0.1 in PCN and ISM media, or OD_600_ ∼ 0.2 in LB broth) or overnight to reach stationary phase (OD_600_ ∼ 0.8, 1.0 and 2.0 in PCN, ISM and LB media, respectively). At these times, 4 ml of culture were added to 1 ml of chilled stop solution (5% phenol in ethanol) [36] and incubated at 4°C for 30 minutes. This step allows bacterial RNA stabilization prior to subsequent treatments [37]. Bacteria were harvested by centrifugation at 4,500× g for 5 min at 4°C, and total RNA was extracted using the Trizol reagent (Life technologies) as described [16]. RNA integrity was confirmed by agarose TAE electrophoresis, and lack of DNA contamination was assessed by lack of amplification products after 30 cycles of PCR with primers OmpA-F and OmpA-R (Table S1). Once confirmed RNA quality and purity, 1 µg RNA were reverse transcribed into cDNA libraries using the High-capacity cDNA archive kit (Life Technologies, Carlsbad, CA). For the synthesis of gene-specific cDNA, 1 µg of total RNA from standing cultures was reverse transcribed for 1 h at 42°C followed by 15 min at 85°C inactivation step with 200 U of the M-MuLV RT (New England Biolabs, Ipswich, MA), 10 pmol of reverse gene-specific primer (Table S1), 4 mM of each dNTP, 2.5 mM MgCl_2_, 6 µg/ml actinomycin D and 0.5 U/ µl RNAse inhibitor. Amplification from gene-specific and cDNA libraries was performed in 1/100 (target) or 1/400 (housekeeping) dilutions of the RT reaction by quantitative real time PCR as detailed below.

### Simultaneous Mapping of 5′- and 3′-ends of RNA Molecules by RACE

Mapping of 5′- and 3′-RNA ends by RACE (Rapid Amplification of cDNA Ends) using circularized RNAs was performed as previously described [8], [38]. Circular RACE involves T4-mediated RNA self-ligation followed an RT-PCR using gene-specific outwards primers. RNA samples were treated or not with the Tobbaco Acid Pyrophosphatase (TAP), which specifically cleaves the pyrophosphate bond of the 5′-terminal resulting in a 5′-monophosphorylated terminus that may be ligated to a 3′-hydroxylated terminus using T4 RNA ligase. This procedure distinguishes primary transcripts, the targets of the TAP, respect processed monophosphorylated RNAs. Thus, primary RNA species are enriched in the TAP− treated RNA fraction as compared to the non-treated when assessing RT-PCR products in RACE assays. The method was used in this work with the following modifications. Briefly, reactions were performed on RNAs extracted from bacteria grown in LB until OD at 600 nm reached a value of 0.2. Six µg of RNA were treated with Turbo DNA free (Life Technologies) to avoid any false positive products. The RNA was divided in two aliquots. Both aliquots were incubated for 1 h at 37°C with the corresponding buffer in presence or absence of TAP (Epicentre Biotechnologies, Madison, WI) respectively. This step allows discriminating a 5′-end generated by transcription initiation from a 5′-end provided by RNA processing. After incubation, acid-phenol and chloroform extractions and ethanol precipitation were performed. Serial dilutions (from 500 ng to 0.5 ng) of the TAP+ and TAP− treated RNAs were prepared. Each dilution were ligated with 40 U of T4 RNA ligase I (New England Biolabs) in the presence of 1×RNA ligase buffer, 8% DMSO, 10 U of RNaseOUT Inhibitor (Life Technologies) and RNase-free water in a total volume of 25 µl at 37°C for 1 h. After acid-phenol and chloroform extractions and ethanol precipitation, the ligated RNAs were resuspended in 10 µl the RNase-free water. RT-PCR reactions were performed using specific outward primers (Table S1) and the One-Step RT-PCR kit (Qiagen, Hilden, Germany). RT-PCR products were checked by loading on a 2% TAE-agarose gels. Bands significantly enriched or only present in the TAP+ reactions were purified by PureLink Quick Gel Extraction kit (Life technologies) and cloned into pGEMT easy (Promega, Madison, WI). Plasmids containing the expected size of insert were sent to sequencing. Sequences were compared with *S.* Typhimurium SL1344 genome (NC_016810) to determine the localization of the 5′- and 3′-ends and subsequently the RNA length.

### Eukaryotic Cell lines and Culture Conditions

Eukaryotic cell lines included NRK-49F normal rat kidney fibroblasts (ATCC CRL-1570), human telomerase reverse transcriptase (hTERT)-immortalized BJ-5ta fibroblasts (ATCC CRL-4001) and HeLa epithelial cells (ATCC CCL-2). NRK-49F fibroblasts were propagated in Dulbecco’s modified Eagle’s medium (DMEM) containing 5% (vol/vol) fetal bovine serum (FBS) and 4 mM L-glutamine. BJ-5ta fibroblasts were propagated in a 4∶1 ratio of DMEM to medium 199 containing 10% FBS, 1 mM sodium pyruvate, and 4 mM L-glutamine. Minimum essential medium Eagle (MEM) containing 10% FBS, 1 mM sodium pyruvate, and 2 mM L-glutamine was used to grow HeLa cells.

### Isolation of RNA from Intracellular *Salmonella*

Bacteria were grown overnight in LB broth at 37°C in non-shaking conditions. Then, bacteria were spun down and washed using Hank’s buffered saline solution (HBSS) prior to infection. An aliquot of these bacteria was used to isolate control RNA from the initial inoculum. For each RNA extraction from intracellular *Salmonella*, a total of 5×10^7^ to 10^8^ fibroblasts or epithelial cells were seeded in 500 cm^2^ culture square dishes (BD Biosciences, ref. 351040) and infected at a multiplicity of 10∶1 (bacteria : eukaryotic cell). Infection was continued for 20 min. Infected cells were washed three times with pre-warmed HBSS and then incubated with fresh culture medium containing 100 µg/ml gentamicin for an additional hour. The media was replaced with fresh medium containing 10 µg/ml gentamicin and infection was continued for as long as indicated. At each time point, infected cells were lysed in 0.1% SDS, 1% acidic phenol, 19% ethanol in RNAse-free water [39]. This phenol-ethanol mixture acted to stabilize bacterial RNA. For each time point, intracellular bacteria were isolated from 2 to 4 culture dishes and pooled. Pellets containing intracellular bacteria were collected by centrifugation at 27,000× g for 30 min at 4°C and washed with phosphate buffered saline (PBS). In some instances, RNA from intracellular bacteria was extracted together with eukaryotic host cell RNA. In these experiments 1 ml of chilled Trizol reagent was added directly to a 100-mm cell culture dish and subsequently scrapped at 4°C. Total RNA was isolated and then reverse transcribed into cDNA libraries as described elsewhere in this section.

### Northern Analyses

Twenty micrograms of total RNA extracted from bacteria grown in standing conditions or from intracellular bacteria were run in 8 M urea 6% acrylamide in 1×TBE buffer for 1 h at RT. Prior to electrophoresis, gel were pre-run for 30 min. RNA was transferred to N^+^Hybond membranes (GE-healthcare) in 0.5×TBE buffer for 2 h at 50V, 4°C. Membranes were UV-cross-linked, pre-hybridized with Ultrahyb-oligo buffer (Life technologies) for 1 h at 42°C. Hybridization was carried out overnight at 42°C in the same buffer containing 1×10^6^ cpms/ml of each of the following primers labelled at their 5′ termini:

*N-0995-1*∶5′-CTGAAAGTACATCAGCAACAGCACTGGCTCTGGGA-3′

*N-0995-2*∶5′-AATCGAAATCCCCCCGACTCGCAATCAGCGCAAATT-3′

*N-0995-3*∶5′-GTGACAATGGGCTCGCATTTATCCTGCCCGGCTCG-3′

*N-0995-4*∶5′-CCGACTGCCAGCATTCTATTTTCCAGTTCGACTGG-3′

Ten pmol of oligonucleotide were labelled with 30 pmol of gamma-^32^P-ATP and using 20 U of polynucleotide-kinase (New England Biolabs) for 30 min at 37°C. Unincorporated nucleotides were separated by spin chromatography (Biospin 6, BioRad) Probe and labelling was quantified by Cerenkov counting. Membranes were washed three times in 2×SSC, 0.5% SDS, at 45°C for 30 min per wash. Membranes were exposed at −70°C for 24 h.

### Quantitative Real-Time PCR

Oligonucleotides were designed using the Primer Express Software (Life technologies) and are listed in Table S1. Reactions were carried out in an ABI Prism 7300 instrument (Life technologies) using the Power SYBR green PCR master mix (Life technologies) under standard reaction conditions described elsewhere [16]. The expression levels of each gene in each condition tested were normalized to the levels of 16S or OmpA transcript.

### Bacterial Infection Assays

Fibroblasts and epithelial cells were seeded in 24-well plates to reach a density of 5×10^4^ to 10^5^ cells/well at the time of infection. Bacteria were incubated overnight without shaking at 37°C in LB broth. Prior to infection, bacteria were washed with pre- warmed HBSS and viable bacteria were enumerated by plating serial dilutions. Cells were infected at a MOI 10∶1 and infection was left to progress 20–30 min until internalized bacteria were observed by microscope examination. Infected cells were washed three times with pre-warmed HBSS and then incubated with culture medium containing 100 µg/ml gentamicin up to 2 h. The media was then replaced with fresh medium containing 10 µg/ml gentamicin and infection was continued for 6 h (HeLa cells) or 24 h (fibroblasts). Cells were lysed at the indicated post-infection times in a solution containing PBS buffer, pH 7.4, 1% (vol/vol) Triton X-100, and 0.1% (wt/vol) SDS. The number of viable intracellular bacteria was calculated by plating appropriate dilutions of the lysate. The invasion rate was the percentage of internalized bacteria calculated by as the ratio between gentamicin-protected bacteria at 2 h after infection and the number of viable bacteria in the inoculum used. The intracellular proliferation index was calculated by the ratio of viable intracellular bacteria in each well at 24 (fibroblasts) or 6 h (HeLa) divided by the viable intracellular bacteria at 2 h post-infection.

### Competitive Index (CI) Virulence Assays

Six-week-old female BALB/c mice (Harlan laboratories, Indianapolis, IN) were used for virulence assays. In each experiment, groups of four to eight animals were inoculated with a 1∶1 ratio of the respective strains. Bacteria were grown overnight at 37°C in LB without shaking, spun down, washed with PBS and diluted. Intraperitoneal inoculation was performed with a 50 µl bacterial suspension containing c.a. 10^5^ viable bacteria. The number of bacteria of each individual strain in the input inoculum was assessed by plating serial dilutions of this suspension in LB and in LB containing the appropriate antibody. To determine the output bacterial load of each strain in target organs, infected mice were sacrificed at 48 to 72 h post-infection, and spleen and liver homogenates were plated. The competitive indexes (CI) were calculated as the ratios in the output samples divided by their ratios in the input inoculum.

### Protein Extracts and Western Blot Analysis

Total protein extracts were prepared from bacteria grown overnight at 37°C without shaking in LB broth or from intracellular bacteria. Bacteria grown overnight in 1 ml of LB broth (∼10^9^ viable bacteria) were collected by centrifugation and suspended in an appropriate volume of Laemmli sample buffer. Intracellular bacteria from infected fibroblasts were collected as described above for RNA isolation. Prior to the comparative protein expression analysis between extracellular and intracellular bacteria, samples were adjusted using either DnaK or OmpA as loading controls. Polyclonal rabbit anti-DnaK and anti-OmpA antibodies have been described elsewhere [23]. Proteins were resolved by Tris-glycine-PAGE using 12% gels and transferred onto polyvinyliden-difluoride (PVDF) membranes using a semidry electrophoresis transfer apparatus (Bio-Rad, Hercules, CA). FLAG-tagged proteins were detected with anti-FLAG M2 monoclonal (1∶5,000 dilution; Sigma, St. Louis, MO). Goat anti-mouse- or anti-rabbit-HRP-conjugated antibodies (Bio-Rad) were used as secondary antibodies (1∶5,000 dilution). Blots were developed with ECL prime reagent (GE Healthcare, Little Chalfont, United Kingdom).

### Accession Numbers

The configuration of the ‘Salgenomics’ microarray was deposited in the MIAME database (http://www.ebi.ac.uk/miamexpress) with accession number A-MEXP-846. Gene expression data were deposited in the Array Express database (http://www.ebi.ac.uk/arrayexpress) with accession numbers E-MEXP-1774 (intracellular *phoP* transcriptome), E-MEXP-1775 (intracellular wild-type transcriptome), and E-MEXP-1776 (extracellular wild-type, stationary phase).

### Statistical Analysis

Prior to carrying out the statistical analyses, relative expression data and ratios coming from infection and virulence assays were fitted to a normal distribution by a log-based transformation. The means of the normalized data were compared by two-tailed student’s t test (two samples), one-sample t-test (one sample *vs.* a hypothetical value) or, by two-way ANOVA and the Bonferroni’s post-test. All data sets were analyzed using GraphPad Prism, version 5.0, software (GraphPad Inc., San Diego, CA).

## Results

### Identification of a Novel Antisense sRNA in the *S.* Typhimurium Virulence Plasmid pSLT

We have recently used the microarray ‘Salgenomics’ for genome expression analyses in intracellular *S.* Typhimurium persisting within fibroblasts [40] and in studies involving extracellular bacteria [16], [23], [30]. This oligonucleotide-based microarray contains specific probes to most known non-coding sRNAs present in intergenic regions (IGRs) [41]. To detect novel sRNAs, the microarray was also designed with a second group of probes positioned in each of the two strands of every IGR along the whole genome. Given our interest in identifying novel sRNAs functionally linked to *Salmonella* infections, we analyzed the expression profile associated to this set of probes in both intracellular and extracellular bacteria. Besides non-growing intracellular bacteria, a sample consisting of bacteria grown to stationary phase in LB broth was included to distinguish transcriptional profiles linked genuinely to the ‘non-proliferative intracellular lifestyle’. Additional information was also obtained by analyzing the profile of the *phoP* mutant overgrowing within fibroblasts. Concerning extracellular *phoP* bacteria, our previous transcriptomic data revealed minimal differences relative to wild-type bacteria when both strain grow actively in LB broth [23]. In all conditions tested, the prolife of extracellular wild-type bacteria growing in LB broth to early-exponential phase was used as common reference sample.

**Figure 1:**
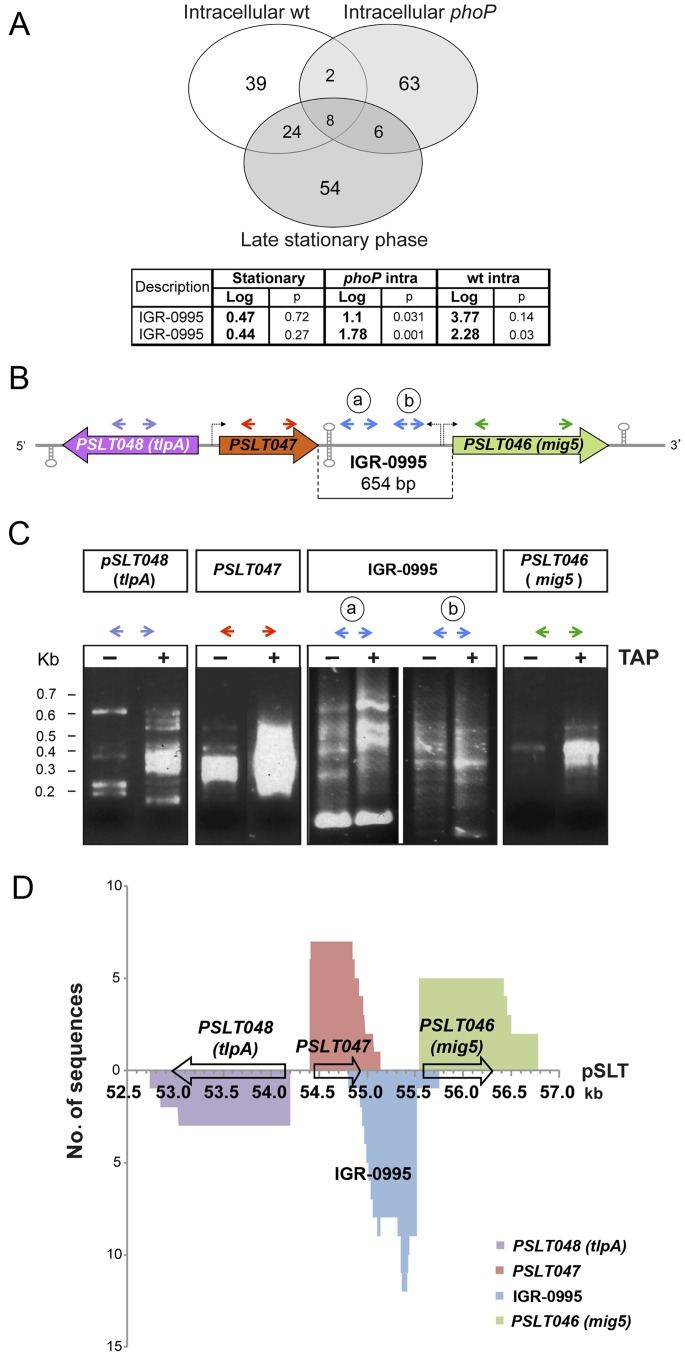
Identification of a novel sRNA in the virulence plasmid pSLT of *S.* Typhimurium. (A) Venn diagrams showing the number of oligonucleotide probes corresponding to intergenic regions (IGR) induced in three experimental conditions (intracellular wild-type, intracellular *phoP*, late stationary phase wild-type) as compared to wild-type bacteria growing in exponential phase of growth (see text and ref. [23] for details). Significance threshold was established at log_2_-expression ratios ≥2.0. The table refers to microarray data of the CNB1344-0995 probe expressed as log_2_ changes in relative levels. The probe was spotted as duplicate in different locations of the microarray slide; (B) Schematic representation of the pSLT plasmid region encompassing the *PSLT048* (*tlpA*)*-pSLT047-PSLT046* (*mig5*) genes. Bended arrows indicate predicted transcriptional start sites of RNAs expressed in this region. Hairpins indicate predicted Rho-independent terminators. Relative positions of the primers used in circular RACE are indicated as colored arrows; (C) Amplification products obtained from circular RACE in pyrophosphatase-treated (TAP+) and control (TAP−) RNA samples. On the left, approximate electrophoretic mobility of molecular weight standards corresponding to 0.7, 0.6, 0.5, 0.4, 0.3 and 0.2 Kb. Results for IGR-0995 correspond to the amplification products obtained using the two primer pairs indicated in the panel B as pairs “a” and “b”; (D) Sequence coverage of clones obtained by circular RACE. Y-axis represents the number of times that a nucleotide in a certain position was found in the sequenced clones. X-axis represents the sequence of the pSLT virulence plasmid, and the coordinates in Kb are in accordance to the NCBI reference sequence NC_017720. Arrows indicate the position of the different coding sequences.

The transcriptomic analyses in non-growing intracellular wild-type bacteria revealed a significant induction (>4-fold, *p*<0.05) in probes mapping to 73 IGRs (Figure 1A). Ten of these 73 probes were also registered with increased signal in intracellular *phoP* overgrowing bacteria and 32 were in common with enhanced signals obtained in bacteria grown to late-stationary phase in LB broth (Figure 1A). Interestingly, 39 transcripts encoded in IGRs were found specifically induced in non-growing intracellular dormant bacteria, which suggests the occurrence of presumptive sRNAs with a putative role in pathogen persistence within fibroblasts (Figure 1A). Among these 39 probes, two of them (CNB1344-0963 and CNB1344-0995) lie to the *Salmonella* virulence plasmid pSLT. The sequence of these two probes in shown as supporting information (Figure S1). At the time of designing the ‘Salgenomics’ microarray, the CNB1344-0963 probe was located within an “empty” IGR non-containing any protein-encoding open reading frame. However, the subsequent comparison to other *S.* Typhimurium plasmids (pSTU288-1, p798_93, pYT2, pSal8934b, pSal6919a, pSTUK-100, TY474p1, pSTMDT12_L, pYT1) indicated that *trbE* gene maps to this region. Since the CNB1344-0963 probe is located between *traN* and *traF* and they are likely to be transcribed as an operon, the transcriptional signal detected from this probe could most probably correspond to the polycistronic mRNA rather than to a novel sRNA. We therefore focused in the transcript detected by the CNB1344-0995 probe (Figure 1A).

The CNB1344-0995 probe maps to the IGR between *PSLT047* and *PSLT046* genes, which hereafter is referred to as IGR-0995 (Figure 1B). These adjacent genes are transcribed in the same orientation and separated by 654 bp (Figure 1B). *PSLT047* encodes an hypothetical 162 aa protein of unknown function, not containing any domain known and with orthologs exclusively in some *S. enterica* serovars. *PSLT046*, also named *mig-5*, was discovered as a gene up-regulated in bacteria located inside macrophages [42]. The PSLT046 protein, of 246 aa, has a transmembrane region and is predicted to contain a ‘carbonic anhydrase’ domain (Pro_CA, Pfam PF00484). Like in PSLT047, the function of PSLT046 remains unknown. Interestingly, the CNB1344-0995 probe was designed to hybridize with a molecule transcribed from the strand complementary to that containing *PSLT046* and *PSLT047*. The genomic organization of this locus therefore rules out the possibility that the detected transcript signal of the CNB1344-0995 probe could derive from a long UTR from the flanking genes. To precisely depict the transcriptional map of the *PSLT047*-*PSLT046*(*mig5*) region, we used a modified version of the method involving Rapid-amplification of cDNAs ends (RACE), so-called ‘circular RACE’, which allows simultaneous identification of 5′ and 3′ ends within the same RNA molecule [8] (Figure 1C, see Material and Methods for details). We found many different transcripts in the *PSLT047*-*PSLT046* (*mig5*) region mainly differing in their 3′ ends (Figure 1D). For the *PSLT046* (*mig5*) gene, most of the sequenced clones corresponded to a 960 nt mRNA while a longer version that spanned until coordinates 56.7 kb of the pSLT plasmid was identified in a few remaining clones (Figure 1D). For the *PSLT047* gene and the putative sRNA encoded in the IGR-0995, several clones whose sequences differed in the 3′ end were characterized, being the longest of these sequences of 740- and 597-nt, respectively (Figure 1D). The majority of the sequenced clones covered the non-overlapping sequence of these two transcripts. In turn, the sequence coverage of the overlapping region in the sequenced clones of both genes (*PSLT047* and that encoding the putative new sRNA) progressively decreased at their respective 3′ ends (Figure 1D). This result could not be ascribed to the processivity of the reverse transcriptase or the natural instability of the 3′-end of bacterial transcripts as longer transcripts could be accurately mapped in the same assay [see PSLT048 (*tlpA*) and *PSLT046* (*mig5*) in Figure 1D]. A closer analysis to the IGR-0995 sequence predicted at the 5′ end a sigma-70 dependent promoter that partially overlaps with the predicted promoter of *PSLT046* (*mig5*) and a stem-loop structure compatible with a Rho-independent terminator at the 3′ end of this putative sRNA (data not shown). Bioinformatics tools as Translate or ORFinder predict in the 597 nt sequence of IGR-0995 two ORFs encoding hypothetical polypeptides of 46 and 52 aa (data not shown). Putative Shine-Dalgarno sites of low score precede these two ORFs and BLAST searches reveal that none of these two polypeptides has homologs in databases (data not shown). Altogether, these analyses supported the existence of an sRNA selectively expressed by non-growing intracellular *S.* Typhimurium inside fibroblasts, which hereafter we named IesR-1, for “Intracellular-expressed-sRNA-1”. The transcriptional map of the *PSLT047-iesR-1-PSLT046* (*mig5*) locus suggests that IesR-1 could act as an antisense sRNA partly overlapping with the *PSLT047* transcript, therefore involving a *cis*-antisense regulatory mechanism [26].

### IesR-1 Expression is Specifically Induced in Non-growing Intracellular Bacteria

**Figure 2:**
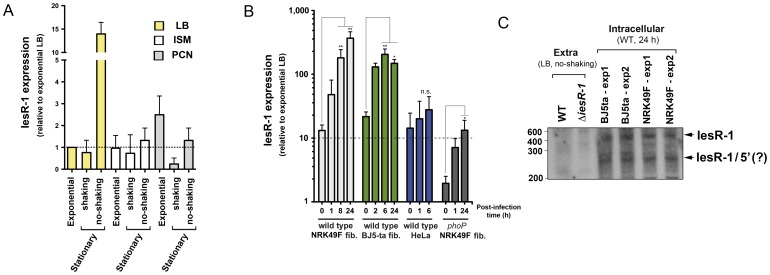
The sRNA IesR-1 is up-regulated in non-growing intracellular *S.* Typhimurium. Relative levels of the sRNA IesR-1 were determined by reverse transcription and RT-qPCR. Data are relative to the transcript levels of IesR-1 in bacteria cultured with shaking in LB broth to early-exponential phase (O.D._600_ ∼0.2). 16S ribosomal RNA was used as a reference gene. Bars indicate the mean ± standard deviation of three independent experiments. (A) IesR-1 expression in axenic cultures. Bacteria were cultured in LB broth and minimal media such as ISM [35] and PCN [34] with shaking to either exponential or stationary growth phases. A third condition consisting in growth with no shaking to stationary phase was also included in the analysis. (B) Expression of IesR-1 in non-growing intracellular *S.* Typhimurium collected at different post-infection times from rat and human fibroblasts (NRK-49F and BJ-5ta, respectively). For comparison, IesR-1 expression was also monitored in wild-type bacteria proliferating inside HeLa epithelial cells and in *phoP* mutant bacteria overgrowing within NRK-49F fibroblasts. The 0 h post-infection time point corresponds to bacteria grown overnight with no shaking in LB broth that were used to infect the eukaryotic cells. Note the pronounced up-regulation of IesR-1 (∼200–300 fold) in non-growing intracellular bacteria at late post-infection times, which contrasts with the moderate induction values registered in extracellular bacteria. Data are the mean and the standard deviation of three independent experiments. (C) Northern blot analysis showing the production of an sRNA of ∼600 nt in intracellular bacteria compatible with the expected size of IesR-1. A second transcript of ∼275 nt was also detected specifically in intracellular bacteria, which as denoted by the RACE experiments (see Figure 1 D) could correspond to 5′ region of IesR-1. Note the lack of noticeable amount of these two molecules in wild-type bacteria grown extracellularly.

Transcriptomic data obtained with the Salgenomics microarray indicated that the *iesR-1* gene is preferentially expressed in non-growing bacteria persisting within fibroblasts. To get insights into the function of this sRNA, we monitored its expression in infection-related conditions. Reverse transcription and quantitative PCR (RT-qPCR) were performed under laboratory culture conditions reported to mimic different stages of the infection process. The intracellular salts medium (ISM) was designed to emulate the cytoplasm of mammalian cells [35] while the PCN defined medium, which has limited amounts of phosphate, carbon and nitrogen, reproduces some of the environmental traits known for the *Salmonella*-containing vacuole [34]. Bacteria were grown under shaking conditions to either exponential or stationary phase in LB broth, ISM or PCN media (Figure 2A). The condition used routinely to grow bacteria for infection of eukaryotic cells, non-shaking, stationary phase in LB broth, was also included. To overcome the bias inherent to the processing at the 3′-end observed in the RACE experiments (see Figure 1D), the primers used for RT-qPCR were designed to amplify a region unique to the 5′ end of IesR-1. Expression of IesR-1 was detected, albeit at very low levels, in LB under shaking conditions (Figure 2A). Consistently with the microarray data (Figure 1A), we did not observe a significant difference in LB at stationary phase compared to exponential phase (Figure 2A). IesR-1 expression was however significantly higher (∼12-fold) in bacteria grown to stationary phase in LB under non-shaking conditions (Figure 2A). Bacteria grown in ISM or PCN media did not induce IesR-1 at significant levels (Figure 2A). Taken together, these results indicate that the high expression levels of IesR-1 inferred by microarray analysis in non-growing intracellular *S.* Typhimurium could not be reproduced in laboratory conditions.

Based on this observation, we next quantified the relative abundance of IesR-1 at different post-infection times upon bacterial entry into eukaryotic cells. NRK-49F rat fibroblasts, the host cells in which the Salgenomics microarray was used to obtain genome expression data of non-growing dormant intracellular bacteria [23], were infected to that purpose. Intracellular bacteria were collected at 1, 8 and 24 h post-infection and the transcript levels measured by RT-qPCR. IesR-1 levels increased notoriously upon infection of NRK-49F cells compared to the bacteria of the inoculum used to infect the fibroblasts (Figure 2B). Such induction became remarkably conspicuous as the infection progressed, showing an increase of ∼300-fold at 24 h post-infection (Figure 2B). Such exacerbated expression of IesR-1 was also observed in non-growing bacteria persisting within BJ5-ta human fibroblasts (Figure 2B). The pronounced expression of IesR-1 in intracellular bacteria was also corroborated by Northern blot analysis, which demonstrated the presence of two IesR-1 transcripts, of ∼600 nt and ∼275 nt, exclusively in samples prepared from intracellular bacteria at 24 h post-infection (Figure 2C). To differentiate whether the up-regulation of IesR-1 in non-growing dormant intracellular bacteria was linked to this particular physiological state of the pathogen or to the host cell type that was infected, IesR-1 expression was monitored in the *phoP* mutant overgrowing in fibroblasts and in wild-type bacteria proliferating within HeLa epithelial cells. In none of these two cases a pronounced up-regulation of IesR-1 as in non-growing intracellular bacteria was observed although the *phoP* mutant produced higher levels of the sRNA (∼5-fold) at 24 h post-infection compared to bacteria present in the inoculum (Figure 2B). This finding led us to discern whether IesR-1 could be subjected to regulation by the PhoP-PhoQ system. We analyzed relative levels of transcript conforming the *PSLT047-iesR-1-pSLT046* (*mig5*) region in wild-type, *phoP* and a *phoP* ^c^ strain carrying a constitutively active PhoP-PhoQ system. While *PSLT046* (*mig5*) was clearly regulated by PhoP-PhoQ, no changes in expression were observed for IesR-1 among the strains tested (Figure S2). Collectively, these data demonstrated the existence of IesR-1 as an sRNA molecule that is selectively up-regulated by *S.* Typhimurium inside eukaryotic cells under conditions in which growth is restrained.

### Role of IesR-1 in *S. Typhimurium* Virulence

Since the expression of many virulence functions of S. Typhimurium has been reported to respond to the intracellular environment, we sought to determine whether an alteration in IesR-1 levels could lead to changes in the capacity of bacteria to invade or persist inside fibroblasts or to impair infection of susceptible mice. To inactivate IesR-1 but simultaneously avoid polar effects on the overlapping 3′ end of *PSLT047* or the 5′ promoter region of *PSLT046* (*mig5*) (see Figure 1A), we replaced a region spanning the first 209 nt of the IesR 5′end with a chloramphenicol-resistance cassette (*cat*) flanked by two FRT sites (Figure S3A). We then checked the expression of the flanking genes as well as the 3′-remnant of IesR-1 using strand-specific primers for reverse transcription followed by RT-qPCR. Wild-type, the aforementioned mutant strain (Δ*iesR-1*/5′::*cat*) and a deletion strain in which the *cat* cassette was eliminated by FLP-mediated recombination of FRT sites (Δ*iesR-1*/5′), were included in this RT-qPCR assay. A polar effect was observed in the Δ*iesR-1*/5′::*cat* strain, as the remaining 3′ region of *iesR-1* was dramatically induced (Figure S3B). This effect was most likely due either to the presence of a cryptic driver in the *cat* cassette or to a read-through of the RNA polymerase as no increased transcription of the 3′ region of *iesR-1* was observed in the clean Δ*iesR-1*/5′ mutant lacking the resistance cassette (Figure S3B). This finding suggested that the natural promoter of *iesR-1* still drives transcription of the remaining of this sRNA that is produced by the clean Δ*iesR-1*/5′ mutant. Since the genomic organization of this plasmid region made not feasible the construction of clean deletion mutants lacking the entire IesR-1 molecule, we included both Δ*iesR-1*/5′::*cat* and Δ*iesR-1*/5′ strains in the infection assays.

**Figure 3:**
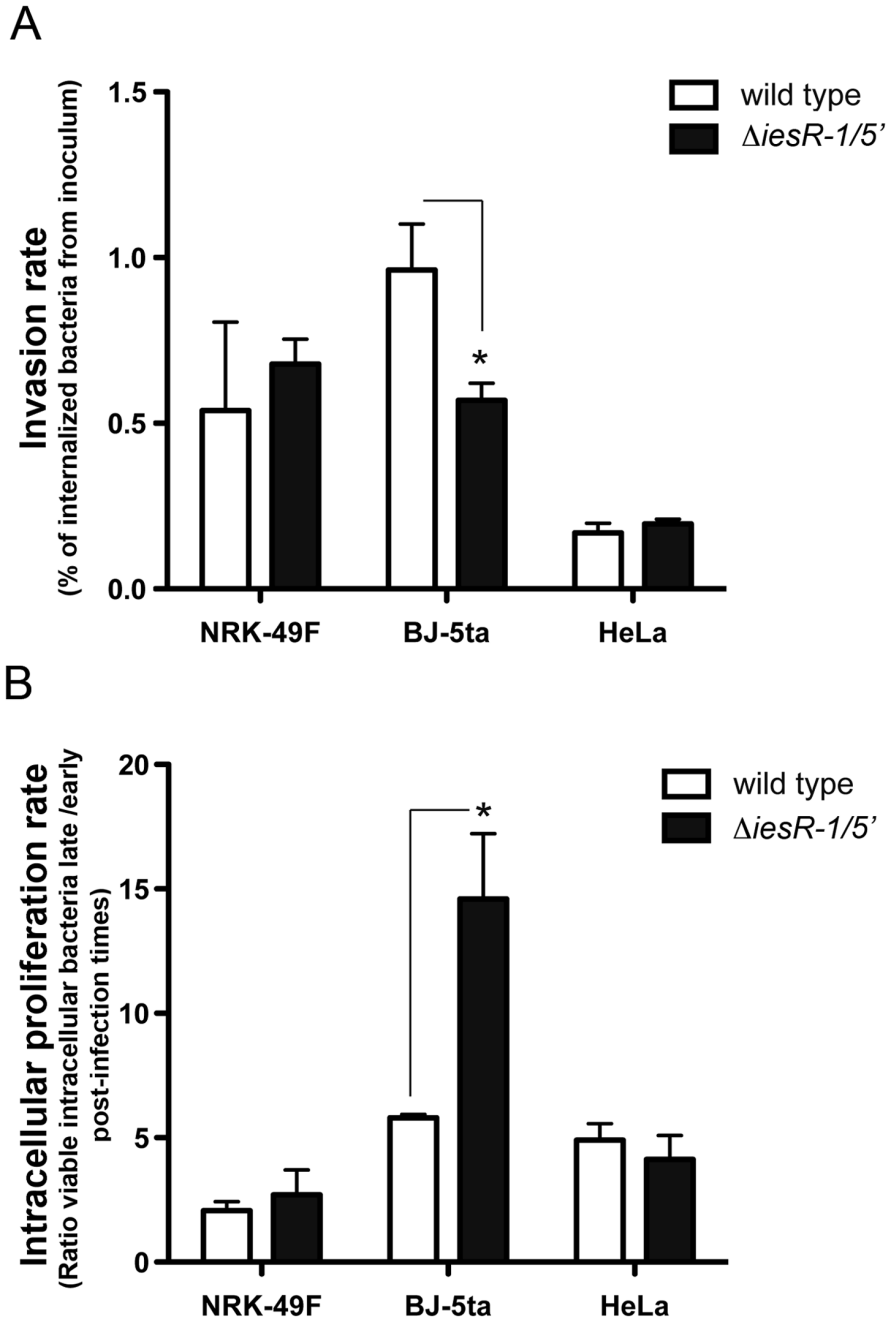
IesR-1 affects the capacity of *S.* Typhimurium to invade and control growth within the human fibroblast cell line BJ-5ta. *S.* Typhimurium wild type (white bars) and its isogenic Δ*iesR-1*/5′ mutant (black bars) were used to infect rat and human fibroblasts (NRK-49F and BJ-5ta, respectively). HeLa epithelial cells were also infected for comparison. Viable intracellular bacteria were counted at 2 h, 6 h (HeLa cells) and 2 h, 24 h (fibroblasts) post-infection. (A) Invasion rates. Bars represent the percentage of bacteria from the initial inoculum that was internalized by the cells upon 30 min of incubation. (B) Intracellular proliferation rates. Bars represent the ratio between the number of viable intracellular bacteria counted at 24 h (fibroblasts) or 6 h (HeLa cells) relative to that determined at 2 h post-infection. Values are the mean ± standard deviation from three independent experiments. (*) *P*<0.05 in student’s t test.

**Figure 4:**
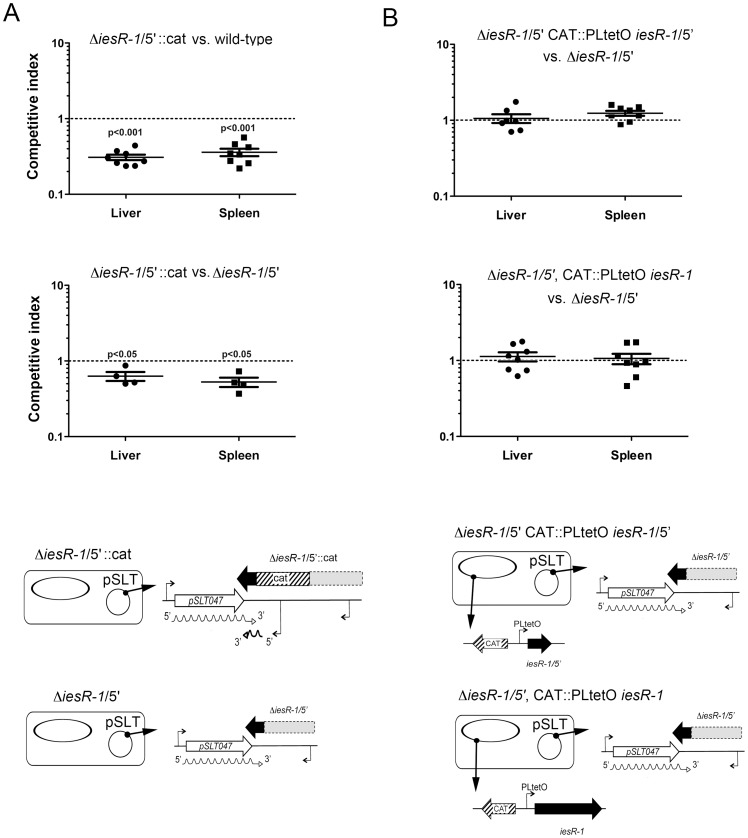
Alteration in the integrity of the IesR-1 molecule causes a defect in virulence that is not restored by complementation in *trans*. (A) Strains Δ*iesR-1/5′*::*cat* or Δ*iesR-1/*5′ were used in competition experiments in susceptible BALB/c mice. While both strains are devoid of the 5′ region of IesR-1, the strain Δ*iesR-1/5′*::*cat* overexpresses the 3′ region (lower part of the panel and Figure S3). Note that both strains are attenuated in virulence; (B) Competition experiments with strains Δ*iesR-1/*5′ overexpressing in *trans* from a construct positioned in the *araE* locus the entire IesR-1 molecule or exclusively the 5′ region (208 nt) of IesR-1 (lower part of the panel). Note that in any case the expression in *trans* of these molecules restored virulence. Groups of four to eight mice were inoculated with the indicated strain pairs by the intraperitoneal route. Liver and spleen were extracted 72 h post-infection and homogenated. Appropriate dilutions were plated onto LB plates with and without antibiotic and the competitive index (CI) calculated as the ratio between strains in the organs versus the ratio in the inoculum. *p*-values were obtained by one-sample student’s t test with log-transformed data and establishing 0 as hypothetical value.

Wild-type and Δ*iesR-1*/5′ strains were used in *in vitro* infection assays with rat and human fibroblasts (NRK-49F and BJ-5ta cell lines, respectively) and human HeLa epithelial cells. Except for BJ-5ta fibroblasts, in which the lack of IesR-1 was linked to a decrease of ∼2-fold in the invasion rate, no significant differences were observed in the rest of cell lines used (Figure 3A). Concerning intracellular phenotypes, the only observed change was also observed in BJ-5ta human fibroblasts, in which the lack of IesR-1 correlated to an increase of ∼3-fold in the intracellular proliferation rate (Figure 3B). Since only minor alterations were observed in the *in vitro* infection models, we tested these strains in the mouse typhoid model. Previous studies reported a strong correlation between *S.* Typhimurium functions required for maintaining a persistent state within fibroblasts and their relative contribution to virulence [43]–[46]. So, we hypothesized a probable role in virulence of IesR-1, strongly up-regulated by non-growing intracellular bacteria (Figure 2B). We then examined the fitness of Δ*iesR-1*/5′::*cat* and Δ*iesR-1*/5′ strains in BALB/c mice using competitive infections upon intraperitoneal challenge. The Δ*iesR-1*/5′::*cat* mutant displayed a decreased fitness compared to wild-type bacteria (competitive index, CI ∼ 0.33) (Figure 4A). This result suggested that either the overexpression of the 3′ region, the lack of the 5′ region, or a combination of both, might attenuate virulence. Similar results were obtained when infecting mice by the oral route (results not shown). Interestingly, the Δ*iesR-1*/5′::*cat* mutant also displayed decreased fitness when mixed with Δ*iesR-1*/5′ mutant bacteria (Figure 4A). Since both strains are devoid of an identical 5′ region of IesR-1, this finding indicates that an increased expression of the 3′ region of IesR-1 might impair virulence. Competition experiments were also performed with the Δ*iesR-1*/5′ strain expressing *in trans* two versions of IesR-1: one encompassing the entire 597 nt molecule, MD2277 (Δ*iesR-1*/5′, *cat*::PLtetO *iesR* ); and, the other carrying only the first 208 nt of the 5′ region, MD2276 (Δ*iesR-1*/5′, *cat*::PLtetO *iesR*-5′). Expression of these IesR-1 molecules was verified by RT-PCR (data not shown). When these two complemented strains were confronted against the Δ*iesR-1*/5′ mutant no changes in virulence were observed (Figure 4B). Altogether, these results indicate that deregulation of IesR-1 expression in *S.* Typhimurium impacts of the progression of the infection and, simultaneously, suggest that the regulation that IesR-1 exerts over the PSLT047 transcript may require interaction of both RNA molecules while they are co-expressed in a specific location of the virulence plasmid.

### IesR-1 Regulates Production of the PSLT047 Protein by a *Cis*-antisense Mechanism

**Figure 5:**
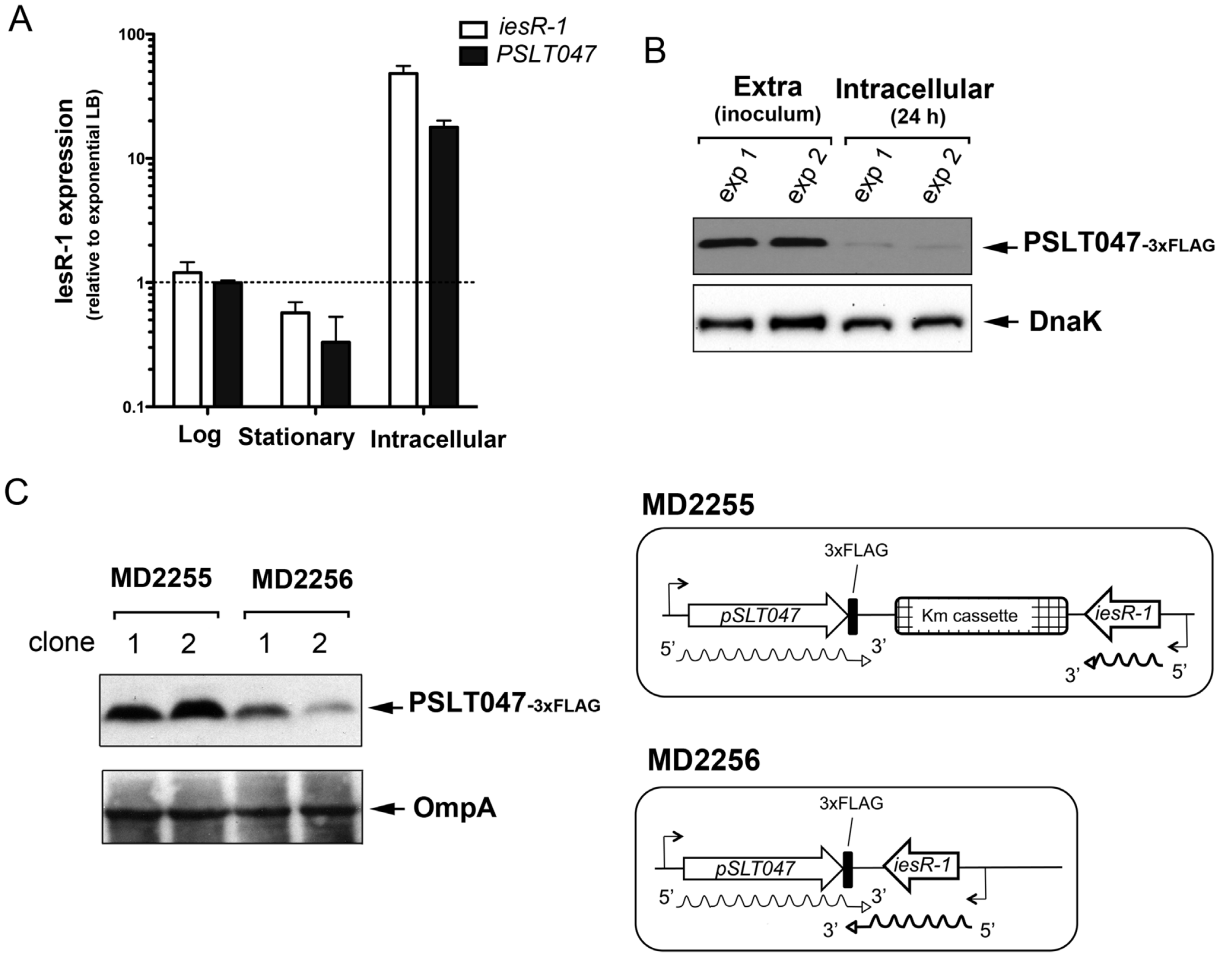
IesR-1 regulates production of the PSLT047 protein by a mechanism involving interaction at the 3′ ends of the respective RNA molecules. (A) expression pattern of *iesR-1* and *PSLT047* in extracellular bacteria grown in LB broth and in intracellular bacteria at 24 h post-infection of NRK-49F fibroblasts. Transcript levels were determined by reverse transcription and RT-qPCR. Expression data were calculated relative to the levels in bacteria cultured to early-exponential phase. 5S ribosomal RNA and the *ompA* transcript were used as endogenous controls for *iesR-1* and *PSLT047* transcripts, respectively. Bars indicate the mean ± standard deviation of three independent experiments. (B) Non-growing intracellular bacteria persisting in fibroblasts down-regulate the levels of the PSLT047 protein. The relative levels of PSLT047 were determined using a PSLT047::3×FLAG-tagged variant. Samples were prepared from bacteria grown in LB broth overnight in no-shaking conditions (inoculum) and from non-growing intracellular bacteria collected from NRK-49F fibroblasts at 24 h post-infection. The results from two independent experiments are shown. (C) Increased production of the PSLT047::3×FLAG-tagged variant in bacteria in which interaction between the 3′ ends of *iesR-1* and *PSLT047* is impeded by the presence of an antibiotic resistance cassette. The genetic configuration of the strains used is indicated. Results from two independent clones are shown. DnaK and OmpA were used as loading controls.

sRNAs that partially overlap with transcripts originated from flanking genes usually exert a *cis*-regulatory mechanism by antisense pairing [26]. Since IesR-1 partially overlaps with the 3′UTR of the *PSLT047* transcript, we hypothesized on a possible antisense regulatory mechanism. Our previous results indicated that IesR-1 is selectively induced in non-growing dormant intracellular bacteria (Figure 2B). An inverse correlation between IesR-1 levels and its putative *cis*-regulated target would suggest a negative post-transcriptional regulation at the level of target mRNA stability. To test this, we monitored the expression pattern of *PSLT047* and *iesR-1* transcripts in different growth conditions (Figure 5A). Unexpectedly, *PSLT047* mRNA expression followed essentially the same induction pattern as its putative sRNA *cis*-acting regulator, IesR-1 (Figure 5A). These results indicated that IesR-1 could have either no influence or, alternatively, a positive effect on PSLT047 mRNA stability. In this scenario, post-transcriptional control at the level of PSLT047 translation could also occur. When analyzed in non-growing intracellular bacteria, we observed that PSLT047 protein levels dropped significantly compared to extracellular bacteria in the inoculum (Figure 5B). Experiments with two strains carrying the *PSLT047*-3xFLAG tagged allele but differing in the integrity of the IesR-1 molecule provided additional support to the model involving interaction between 3′ regions of IesR-1 and the *PSLT047* transcript with effects in PSLT047 protein levels. Thus, the amount of PSLT047 increased significantly in extracellular bacteria having an IesR-1 molecule interrupted with a Km resistance cassette, therefore unable to interact at its 3′ end with the *PSLT047* transcript (Figure 5C). Altogether, these results are consistent with a *cis*-acting regulatory mechanism involving interaction of the antisense IesR-1 molecule with the *PSLT047* mRNA, which could ultimately modulate the translation rate of this protein.

## Discussion

Computational, experimental and global RNAseq approaches have been used to search for non-coding regulatory sRNAs in *S.* Typhimurium [6], [7], [17], [47]. These studies have been mostly restricted to bacteria grown *in vitro* conditions, providing little insights into the transcription status during *ex vivo* or *in vivo* infections. A recent work in the mouse typhoid model in which *S.* Typhimurium mutants lacking defined sRNAs were used, uncovered the requirement of IstR, OxyS and SroA for virulence [15]. Additional studies showed that the sRNAs IsrJ and IsrM contribute to invasion of epithelial cells and that *S.* Typhimurium also uses IsrM for proliferation in mouse organs [11], [17]. Apart from these studies, most sRNAs identified to date in *S.* Typhimurium remain to be shown whether they play a role in virulence [48].

In this work, we focused on transcriptional changes in *S.* Typhimurium persisting within fibroblasts, a host cell type in which intracellular bacteria establish a non-proliferative, dormant state [24]. Persistence is a strategy used by successful pathogens, including *S. enterica*, *M. tuberculosis,* or *Helicobacter pylori* among others [21]. Our previous work demonstrated that certain *S.* Typhimurium sRNAs encoded in the chromosome exhibit unique expression pattern along the fibroblast infection [16]. This observation is indicative of distinct time-dependent physiological roles for these molecules during progression of the persistence state. Here, we analyzed a novel *S.* Typhimurium sRNA encoded in the pSLT virulence plasmid that we named IesR-1. Besides FinP, IesR-1 would be the second example of a pSLT-encoded sRNA that, in this case, could have evolved to modulate the growth rate of the pathogen inside the eukaryotic cell. Interestingly, other functions encoded in the pSLT plasmid, such as the transcriptional regulator SpvR, are induced in a *Caenorhabditis elegans* persistent infection model [49]. Intriguingly, *spvR* mutants are defective in their capacity to restrain growth inside fibroblasts [24]. Although pSLT is known to be required for *Salmonella* virulence, little is known about additional regulatory molecules encoded by this plasmid playing a role in pathogen persistence.

Our finding that the sRNA IesR-1 is specifically induced during persistence (Figures 1–2) highlights the importance of using physiological models to identify novel virulence factors. Indeed, we failed to detect up-regulation of IesR-1 in extracellular bacteria growing in either ISM or PCN defined media, considered to mimic the conditions of the vacuole inhabited by intracellular bacteria. This result indicates that intracellular signals resulting from host cell-microbe interaction entail a higher level of complexity, and that such a particular condition could be hardly accomplished by current *in vitro*-culturing reagents. The persistence-dependent expression of IesR-1 was confirmed in fibroblast from different sources (human and rat origin) although it was not so evident in *phoP* mutant bacteria actively proliferating within host cells (Figure 2B). These results open the question of whether IesR-1 expression is regulated in response to specific signal(s) implicated in quorum sensing and/or adaptive responses linked to environmental stresses existing in the *Salmonella*-containing vacuole.

The identification of IesR-1 as a novel sRNA was confirmed by circular RACE experiments and further cloning of amplicons enriched in the pyrophosphatase-treated fractions as well as Northern blot. The RACE experiments allowed to accurately map the 5′-end of the primary transcripts. Regarding the 3′ end, the transcripts showed a marked heterogeneity among the sequenced clones, which is likely to result from read-through of transcriptional terminators and/or degradation by 3′-exonucleases. This is a particularly prominent feature in the overlapping region between *PSLT047* and *iesR-1* transcripts, for which we got clones with gradually decreasing lengths. This result could indicate an RNase III-mediated post-transcriptional endonucleolytic processing of an hypothetical *PSLT047*-*iesR-1* duplex. Alternatively, such heterogeneity in the 3′ ends could also be explained by the fall off of the reverse transcriptase as a result of encountering dsRNA tracks with variable size made of the *PSLT047-iesR-1* hybrid RNAs. Regardless of the situation, our results are consistent with a *cis*-acting regulation of IesR-1 over the *PSLT047* transcript by an interaction of their respective 3′-ends. This postulate is also sustained by the increased production of the PSLT047 protein following the interruption of the IesR-1 molecule (Figure 5C). Such convergent genetic configuration between an sRNA and the 3′ UTR of its target has been previously documented. Thus, the GadY sRNA of *Escherichia coli* overlaps with the 3′UTR of GadX, a transcriptional regulator of acid response. This interaction leads to an RNase III-mediated processing of the GadX-GadW duplex, which in turn favors the accumulation of GadX transcript [50]. Conversely, the *Bacillus subtilis* RatA sRNA overlaps with the 3′UTR of *txpA*, which codes for a toxic peptide that promotes cell lysis [51]. This interaction impedes *txpA* transcript accumulation, thus avoiding cell lysis. Nevertheless, the data obtained with IesR-1 are more consistent with a regulatory mode over PSLT047 acting on its translation rate.

The presence of antisense transcripts in mobile genetic elements (such as plasmids) is frequently associated to type I toxin-antitoxin systems (TAS) [26], [27]. These systems contribute to maintain stability of plasmids during bacterial propagation by the post-segregational killing of plasmid-free daughter bacteria. Interestingly, the 3′ end of IesR-1 shows high homology to an intergenic region (Sbal223_609 to Sbal223_610) from *Shewanella baltica* (Figure S4), in which Sbal223_609 is annotated as a putative toxin. This observation suggests a possible horizontal gene transfer between *Shewanella baltica* and *S. enterica* and raises the tempting idea of a possible toxin-antitoxin system conformed by PSLT047 and IesR-1. Such a scenario is reminiscent to the *par* stability determinant of *Enterococcus faecalis* encoded in the pAD1 plasmid [52], [53]. RNA I and RNA II are two sRNAs transcribed in opposite orientations that constitute the toxin and antitoxin of the *par* post-segregational killing system, respectively. Interestingly, the interaction of RNA I and II involves the binding at the 5′ and 3′ ends in a two-step mechanism, with an initial kissing interaction between the transcriptional terminator stem-loops of both RNAs followed by the pairing of the complementary direct repeat sequences and the complete hybridization of the 5′ nucleotides to stabilize the complex [53]. The binding of RNA II antitoxin has been shown to inhibit ribosome binding to RNA I, thus impeding an efficient toxin translation initiation and cell killing [52]. In this regard, it would be of interest to investigate whether IesR-1 could control negatively PSLT047 translation by a similar mechanism. Moreover, we cannot discard the possibility that IesR-1 could regulate expression of other genes located elsewhere in the chromosome and/or the pSLT plasmid *via* a *trans*-antisense mechanism based on the non-overlapping 5′ region. Transcriptomics performed in wild-type and Δ*iesR-1*/5′ strains growing in the most optimal extracellular condition for IesR-1 expression, non-shaking-stationary phase in LB broth, did not result in any conclusive target (data not shown). This observation could imply that the 5′ moiety of IesR-1 does not play a significant role in determining target specificity. On the other hand, it cannot be ruled out that IesR-1 does exert its function exclusively in intracellular bacteria, therefore with no possibility of target identification in extracellular bacteria. Alternatively, IesR-1 might influence translation of pre-existing mRNAs. Similar comparative transcriptomics in intracellular bacteria might provide clues on these aspects, however such study means a real technical challenge, as purification of enough total RNA from two independent strains staying in a dormant non-growing state within the fibroblast requires a large effort. Given our interest in deciphering the biological role of IesR-1, we are currently considering this possibility.

Lastly, the data obtained in the *in vitro* and *in vivo* infection models reveal a feature repeatedly observed for many sRNAs. The lack of the regulatory molecule may result in minor alterations in the capacity of the pathogen to cause infection due to the fine-tuning that most of these sRNA molecules perform in the activity or relative level of their respective targets [54], [55]. Some sRNAs ‘share’ targets, so the absence of a particular sRNA can be compensated by the action of other sRNAs [3]. In the case of IesR-1, our data allow to tentatively assign relevance to a probable coordinated production of ‘both’, the antisense IesR-1 and the PSLT047 transcript. The overexpression of the 3′ region of IesR-1 or the expression in trans of the entire IesR-1 molecule led to negative phenotypes consistent with such idea. Further studies could consider the tempting hypothesis of a coordinated expression of both RNA molecules by intracellular bacteria leading subsequently to an inverse correlation between PSLT047 protein levels and IesR-1 expression. The PSLT047-IesR-1 pair could, in this regard, behave as a type I toxin-antitoxin system resembling others implicated in bacterial growth arrest and persistence [56], [57].

## Supporting Information

Figure S1 **Sequences (5′–3′) of the oligonucleotide probes CNB1344-0963 and CNB1344-0995 mapping in IGRs of the** ***S.*** **Typhimurium pSLT virulence plasmid.** These two probes revealed increased transcriptional activity in their respective IGRs in non-growing intracellular bacteria located inside fibroblasts.
(DOCX)

Figure S2 **The non-coding sRNA IesR-1 is not regulated by the PhoP-PhoQ system.** RT quantitative PCR (RT-qPCR) assays performed on *iesR-1* and its flanking genes in wild-type bacteria (14028 s), an isogenic *phoP*::Tn*10*, and constitutively active *phoP-24* mutant. Expression data were calculated relative to the levels in wild type bacteria. 16S ribosomal RNA was used as endogenous control gene. Bars indicate the mean ± standard deviation of three independent experiments. *, p<0.05 obtained by one-sample student’s t test with log-transformed data and establishing 0 as hypothetical value
(TIF)

Figure S3 **Δ** ***iesR-1*** **/5′ mutants construction and expression analysis of flanking genes.** (A) Schematic representation of the PSLT region containing the *PSL047-PSLT046(mig5)* loci in Δ*iesR-1*/5′ mutant strain. The gray box indicates the deleted region of *iesR* (Δ*iesR*-1/5′). In the Δ*iesR-1*/5′::cat mutant, this region is replaced by a cloramphenicol resistance (*cat*) cassette flanked by two FLP recombinase sites (FRT). In the in Δ*iesR-1*/5′ mutant the cloramphenicol resistance is lost by FLP-mediated recombination (see also Figure 4 main text). Bended arrows indicate the predicted transcriptional start sites of RNAs expressed in this region. Dotted arrows indicate the length and orientation of transcripts identified by RACE; (B) Expression of flanking genes and of the remaining 3′-moiety of *iesR-1* in the Δ*iesR-1*/5′ and Δ*iesR-1*/5′::cat mutants. Expression levels were determined by strand-specific reverse transcription using reverse gene-specific primers, followed by qPCR. Data were calculated relative to the levels in wild type bacteria, and normalized by the geometric mean of 16S, *ompA* and *rnpB* endogenous control genes. Bars indicate the mean ± standard deviation of three independent experiments. ***, p<0.001 as compared to wild type by student’s t test.
(TIF)

Figure S4 **Analysis of IesR-1 orthologs in other bacteria.** Diagram showing regions in the pSLT plasmid of *S.* Typhimurium strain LT2 or the *Shewanella baltica* OS223 chromosome with high homology to the non-coding RNA sequence.
(TIF)

Table S1 **Oligonucleotide primers used in this study.**
(DOCX)
