# Supplementary material for: A Novel Antisense RNA from the Salmonella Virulence Plasmid pSLT Expressed by Non-Growing Bacteria inside Eukaryotic Cells
Source: PLoS One. 2013 Oct 31;8(10):e77939. doi: 10.1371/journal.pone.0077939 (PMC3815029; doi:10.1371/journal.pone.0077939)
Supplement: Figure S1 — Sequences (5′–3′) of the oligonucleotide probes CNB1344-0963 and CNB1344-0995 mapping in IGRs of the S. Typhimurium pSLT virulence plasmid. These two probes revealed increased transcriptional activity in their respective IGRs in non-growing intracellular bacteria located inside fibroblasts. (DOCX) [file pone.0077939.s001.docx]

**CNB1344-0963:**

ATGTCAGGCTCCTGACGAACCTGAGCGGATTTATCCGTCGTTTTTGGGTCAGGTAATTTTACCGGGTCGT

**CNB1344-0995:**

GCAAGTCAACTTCGTCATATCGGCAAACCGACTGCCAGCATTCTATTTTCCAGTTCGACTGGTCTCAGAT
