# Supplementary figures and images for: A Novel Antisense RNA from the Salmonella Virulence Plasmid pSLT Expressed by Non-Growing Bacteria inside Eukaryotic Cells
Source: PLoS One. 2013 Oct 31;8(10):e77939. doi: 10.1371/journal.pone.0077939 (PMC3815029; doi:10.1371/journal.pone.0077939)

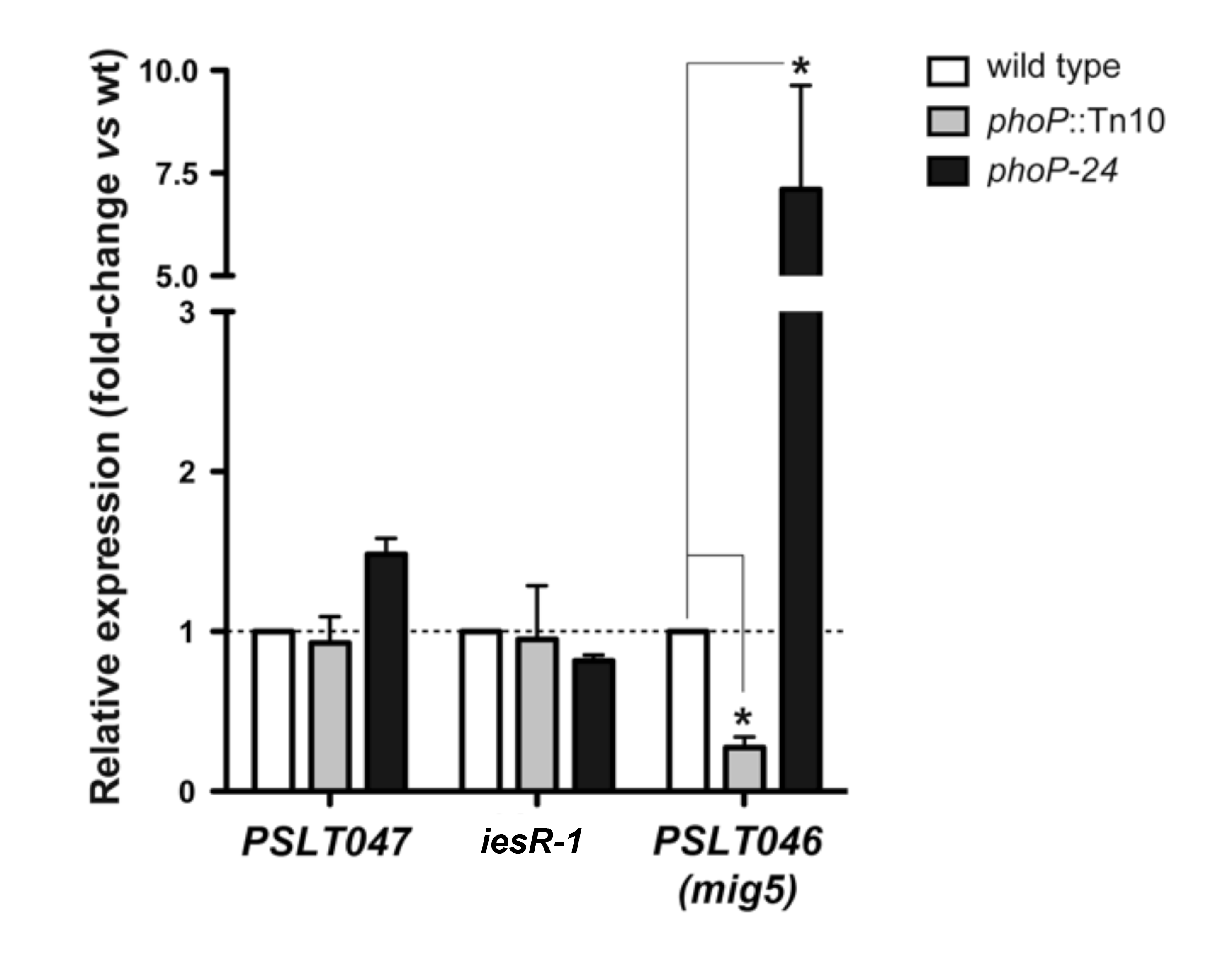

Supplement: Figure S2 — The non-coding sRNA IesR-1 is not regulated by the PhoP-PhoQ system. RT quantitative PCR (RT-qPCR) assays performed on iesR-1 and its flanking genes in wild-type bacteria (14028 s), an isogenic phoP::Tn10, and constitutively active phoP-24 mutant. Expression data were calculated relative to the levels in wild type bacteria. 16S ribosomal RNA was used as endogenous control gene. Bars indicate the mean ± standard deviation of three independent experiments. *, p<0.05 obtained by one-sample student’s t test with log-transformed data and establishing 0 as hypothetical value (TIF) [file pone.0077939.s002.tif]

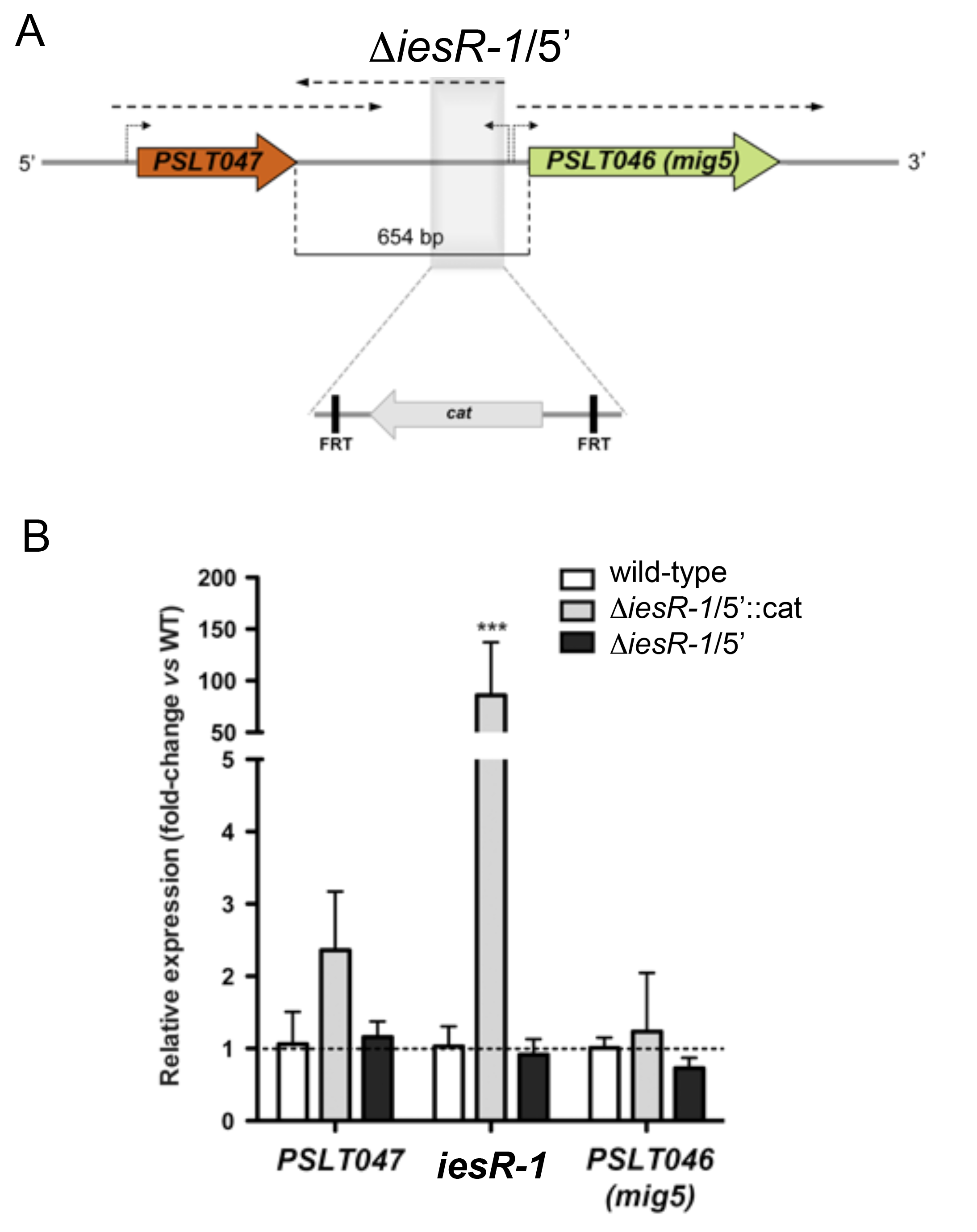

Supplement: Figure S3 — Δ iesR-1 /5′ mutants construction and expression analysis of flanking genes. (A) Schematic representation of the PSLT region containing the PSL047-PSLT046(mig5) loci in ΔiesR-1/5′ mutant strain. The gray box indicates the deleted region of iesR (ΔiesR-1/5′). In the ΔiesR-1/5′::cat mutant, this region is replaced by a cloramphenicol resistance (cat) cassette flanked by two FLP recombinase sites (FRT). In the in ΔiesR-1/5′ mutant the cloramphenicol resistance is lost by FLP-mediated recombination (see also Figure 4 main text). Bended arrows indicate the predicted transcriptional start sites of RNAs expressed in this region. Dotted arrows indicate the length and orientation of transcripts identified by RACE; (B) Expression of flanking genes and of the remaining 3′-moiety of iesR-1 in the ΔiesR-1/5′ and ΔiesR-1/5′::cat mutants. Expression levels were determined by strand-specific reverse transcription using reverse gene-specific primers, followed by qPCR. Data were calculated relative to the levels in wild type bacteria, and normalized by the geometric mean of 16S, ompA and rnpB endogenous control genes. Bars indicate the mean ± standard deviation of three independent experiments. ***, p<0.001 as compared to wild type by student’s t test. (TIF) [file pone.0077939.s003.tif]

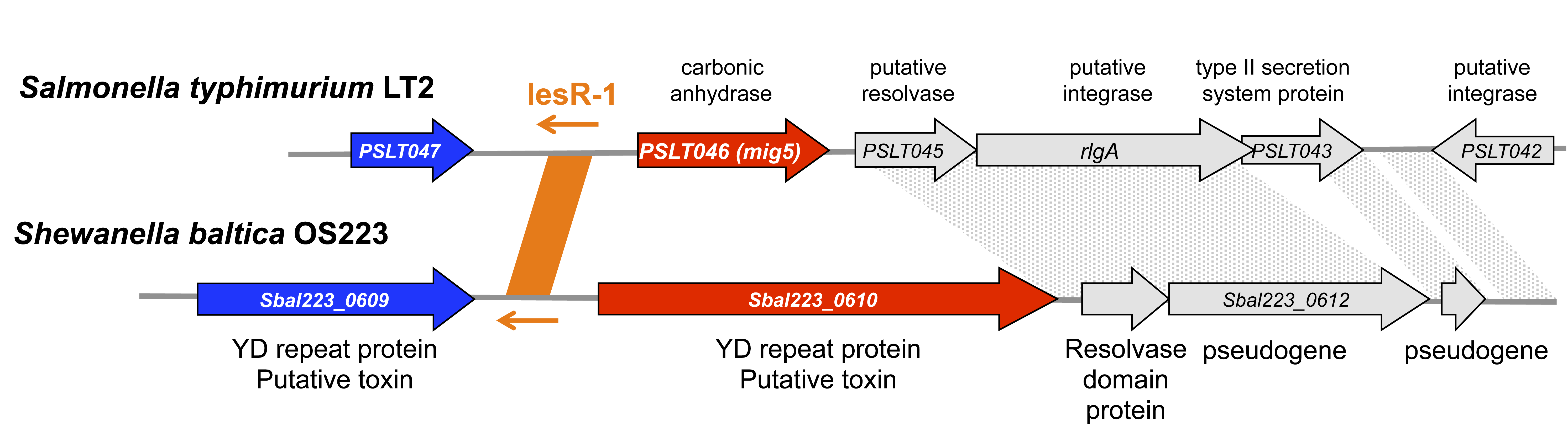

Supplement: Figure S4 — Analysis of IesR-1 orthologs in other bacteria. Diagram showing regions in the pSLT plasmid of S. Typhimurium strain LT2 or the Shewanella baltica OS223 chromosome with high homology to the non-coding RNA sequence. (TIF) [file pone.0077939.s004.tif]
