# Supplementary material for: A Novel Antisense RNA from the Salmonella Virulence Plasmid pSLT Expressed by Non-Growing Bacteria inside Eukaryotic Cells
Source: PLoS One. 2013 Oct 31;8(10):e77939. doi: 10.1371/journal.pone.0077939 (PMC3815029; doi:10.1371/journal.pone.0077939)
Supplement: Table S1 — Oligonucleotide primers used in this study. (DOCX) [file pone.0077939.s005.docx]

**Table S1**. Oligonucleotide primers used in this study

| **Primer name** | **Sequence (5' to 3')** | **Use** |
| --- | --- | --- |
| *KO-995-fw* | TATTTACTGAGTTAACTCCAATTGCTGCGATTATACGTAGCATATGAATATCCTCCTTAGT | Gene inactivation |
| *KO-0995-rv* | CGCAATCAGCGCAAATTTCGGCTCACATTAACTGCAAATGGTGTAGGCTGGAGCTGCTTC | Gene inactivation |
| *FLAG-PSLT047-fw* | CTACCAGAAAAATCAGAACAGACAGAAAGGCGCAGGGGATGACTACAAAGACCATGACGG | Epitope tagging |
| *FLAG-PSLT047-rv* | TTAATGCTAATGTACGCATACATTAGCATTAATCATGCATCATATGAATATCCTCCTTAG | Epitope tagging |
| *RACE-0995-1* | GTGAGCCCGGTGGAATGGAT | RACE mapping of the intergenic transcript |
| *RACE-0995-2* | TCAACTTCGTCATATCGGCAAA | RACE mapping of the intergenic transcript |
| *RACE-SO144A-1* | GGCTTGAACGTGGCCATCCC | RACE mapping of the intergenic transcript |
| *RACE- SO144A-2* | AGACCAGACTGAGAGCCACA | RACE mapping of the intergenic transcript |
| *RACE PSLT047 3* | ATCTTTTCGACAGGCAGGGC | RACE mapping of the putative PSLT047 gene |
| *RACE PSLT047 4* | CCCAGGTGTCATCCCTGTAGT | RACE mapping of the putative PSLT047 gene |
| *RACE mig5 3* | CTTTGCTCAGTGATGCGGC | RACE mapping of the mig5 gene |
| *RACE mig5 4* | ACGGAGTATAGCGGTGAGCG | RACE mapping of the mig5 gene |
| *qRT-0995-2-fw* | AACGACAATTTTTAACCAGTATGAATAAAT | qPCR |
| *qRT-0995-2-rv* | TTCCAGTTCGACTGGTCTCAGAT | qPCR |
| *qRT-mig5-fw* | GAGTTTGCCTGTGCAGTTGC | qPCR |
| *qRT-mig5-rv* | GTGTCCTATAACCAGCACTACTTTCG | qPCR and strand-specific RT |
| *qRT-PSLT047-fw* | AACGGGCCAGGGAATACTTT | qPCR |
| *qRT-PSLT047-rv* | GGTCTTCCTCTGTATACCGGTGG | qPCR and strand-specific RT |
| *RT-0995-3’-fw* | CATTCTTTGAGTGTCCGTTTGG | qPCR |
| *RT-0995-3’-rv* | CAACGTTGCGAAGGTTCTTTG | qPCR and strand-specific RT |
| *16s-F* | CCTGGGAACTGCATTCGAA | qPCR (control) |
| *16s-R* | TGGAATTCTACCCCCCTCTACA | qPCR (control) |
| *5S-F* | CCCATGCCGAACTCAGAAGT | qPCR (control) |
| *5S-R* | GGGAGACCCCACACTACCAT | qPCR (control) |
| *ompA-F* | TGTAAGCGTCAGAACCGATACG | Detection of genomic DNA and qPCR |
| *ompA-R* | GAGCAACCTGGATCCGAAAG | Detection of genomic DNA and qPCR |
| *PLtetO-0995-FW* | ACTGGTTAAAAATTGTCGTTTTGGTTTGATTAACGTTTACGTGCTCAGTATCTCTATCACTGATAG | Construction of complemented strains |
| *PLtetO-0995-RV* | TATTTACTGAGTTAACTCCAATTGCTGCGATTATACGTAGAGGCTTACCCGTCTTACTGTC | Construction of complemented strains |
| *araE-PLtetO-0995-UP* | TGGGTTTAACTTAATCCATATATTGTTAAATAATAGCTATAGGCTTACCCGTCTTACTGTC | Construction of complemented strains |
| *araE-PLtetO-0995-short-DOWN* | TTAATAGAGACCATATTTTCCTGCCACAACAGAGTAAGACAAAAATGCCACCGGCTCACA | Construction of complemented strains |
| *araE-PLtetO-0995-long-DOWN* | TTAATAGAGACCATATTTTCCTGCCACAACAGAGTAAGACTGATTAATGCTAATGTATGCG | Construction of complemented strains |
| *araE-UP* | TGGGTTTAACTTAATCCATATATTGTTA | Confirmation of complemented strains |
| *araE-DOWN* | TTAATAGAGACCATATTTTCCTGCC | Confirmation of complemented strains |
|  |  |  |
